# Sialic Acid Metabolic Engineering of Breast Cancer Cells Interferes with Adhesion and Migration

**DOI:** 10.3390/molecules25112632

**Published:** 2020-06-05

**Authors:** Manimozhi Nagasundaram, Rüdiger Horstkorte, Vinayaga Srinivasan Gnanapragassam

**Affiliations:** Institute for Physiological Chemistry, Medical Faculty, Martin-Luther-University, Halle-Wittenberg, 06120 Halle (Saale), Germany; manimozhi.nagasundaram@medizin.uni-halle.de (M.N.); ruediger.horstkorte@medizin.uni-halle.de (R.H.)

**Keywords:** sialic acid, polysialic acid, breast cancer, glycoengineering, sialic acid precursors

## Abstract

Breast cancer is the most frequent cancer diagnosed in women and the second most common cancer-causing death worldwide. The major problem around the management of breast cancer is its high heterogeneity and the development of therapeutic resistance. Therefore, understanding the fundamental breast cancer biology is crucial for better diagnosis and therapy. Protein sialylation is a key posttranslational modification of glycoproteins, which is also involved in tumor progression and metastasis. Increased expression of sialic acids (Sia) can interfere in receptor–ligand interactions and might protect tumor cells from the immune system. Furthermore, Sia content on the cell membrane plays a role in cancer resistance towards chemo- and radiation therapy. In this study, we glycoengineered MCF-7 breast cancer cells using a series of non-natural Sia precursors, which are prolonged in their acyl side chain. We observed a significant reduction in the natural Sia (*N*-Acetylneuraminic acid) expression after cultivation of MCF-7 cells with these Sia precursors. In addition, the expression of polySia, a unique glycosylation of the neural cell adhesion molecule NCAM, which interferes with cell adhesion, was decreased. We conclude that sialic acid engineering i) opens up novel opportunities to study the biological role of Sia in breast cancer and ii) provides a toolbox to examine the sialic acid-dependent complex cellular alterations in breast cancer cell biology.

## 1. Introduction

The primary concern with breast cancer management is the high heterogeneity and variable sensitivity towards standard therapeutic options such as chemotherapy or radiation. Primary breast cancer subtypes are classified based on the expression of estrogen receptor, progesterone receptor or human epidermal growth factor receptor 2 (Her2/neu) [1,2]. Protein sialylation plays a significant role in regulating cellular properties by interfering the function of the associated proteins. Altered sialylation status is associated with cancer since sialylation of glycoproteins regulates the cellular properties such as cell–cell and cell–matrix adhesion, migration of cells, responding towards extracellular stimuli and interaction with immune cells [3]. Breast cancer cells use the various roles of sialic acid (Sia) for their progression and metastasis [4,5].

**Figure 1:**
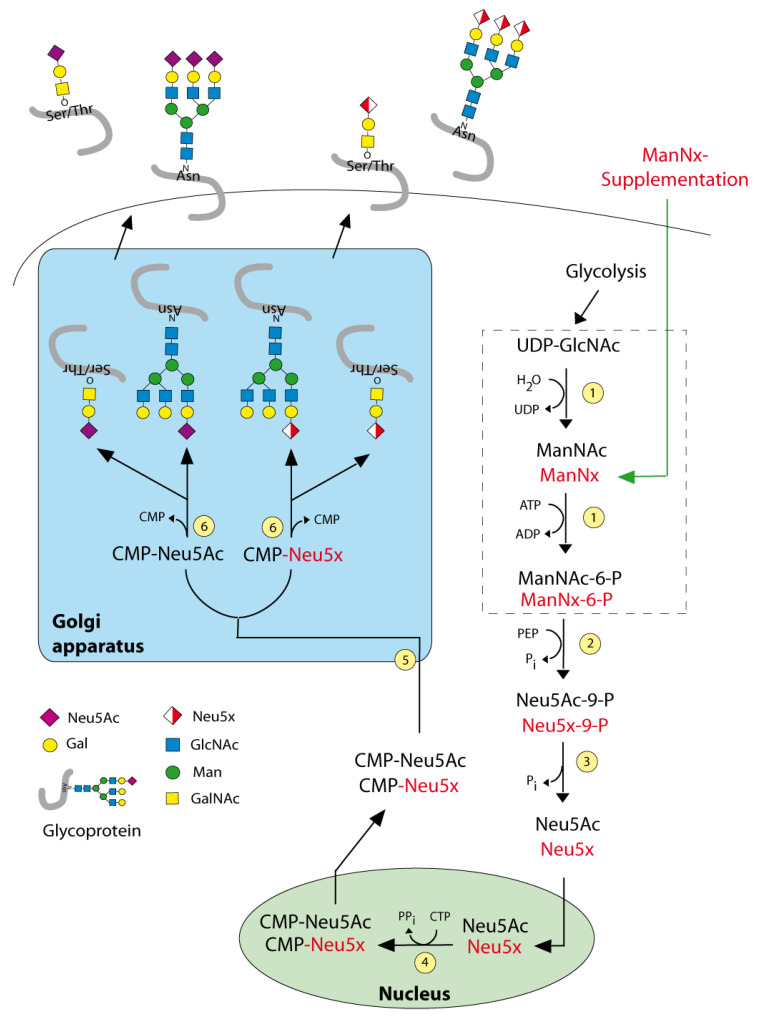
Sialic acid biosynthesis. Enzymes: (1) UDP-GlcNAc 2—epimerase/ManNAc kinase (GNE); (2) *N*-acetylneuraminic acid synthase (NANS), (3) *N*-acylneuraminate-9-phosphatase (NANP), (4) CMP–sialic acid synthetase (CMAS), (5) SLC35A1—CMP-sialic acid transporter, (6) various sialyltransferases. ManNx— mannosamine with x referring to either *N*-proponoyl, *N*-butanoyl, *N*-pentanoyl or *N*-hexanoyl).

Sia represents a 9-carbon sugar family with more than 80 members among them, N-acetylneuraminic acid (Neu5Ac) being the most frequent member of humans [6,7]. Sia is biosynthesized in cytosol using N-acetylmannosamine (ManNAc) and is activated by cytidine monophosphate (CMP) in the nucleus, followed by the addition of Sia to glycoproteins by sialyltransferases in the Golgi apparatus [8] (Figure 1). Expression of Sia is often upregulated in breast cancer [9]. Sia shields the cancer cells from immune surveillance by masking tumor epitopes and thereby reduces the antitumor immunity and alternative complement activation [10]. Furthermore, Sia is involved in the regulation of immune cells by binding to receptors of the SIGLECS (sialic-acid-binding immunoglobulin-like lectins) family of immune effector cells such as NK and T cells by inducing inhibitory tyrosine motifs [11,12,13]. Due to its negative charge, Sia has a role in the negative charge repulsion between cells, which can alter the biophysical properties of cellular interactions and can influence the ability of the metastatic cancer cells to disseminate from the primary tumor. As soon as metastatic cancer cells reach the metastatic sites, Sia can act as ligands for selectins and aid the metastatic cancer cells to bind to the target site [14]. Thus, Sia could play a role in (breast) cancer pathogenesis.

As Sia is essential for the normal cellular function, targeting Sia is a strategically challenging approach; for example, a complete blockade of Sia biosynthesis in vivo leads to lethality [15]. Sialylation is performed with the help of more than 20 different sialyltransferases and 4 sialidases. Their expression is considered as the regulatory step for the sialylation. The biosynthesis of Sia is initiated by converting uridine diphosphate *N*-acetylglucosamine (UDP-GlcNAc) to ManNAc by the Sia master regulator GlcNAc epimerase/ManNAc kinase (GNE). The application of 5-acyl-modified mannosamines with the propanoyl groups was tolerated by the Sia biosynthetic pathway and it is able to generate non-natural Sia [16,17,18]. We extended these studies in the neuroblastoma cell line by glycoengineering with *N*-propanoyl (ManNProp) and *N*-pentanoylmannosamines (ManNPent), which diminished migration and invasion [19]. In this study, we synthesized the following non-natural sialic acid precursors: *N*-propanoylmannosamine (ManNProp), *N*-butanoylmannosamine (ManNBut), *N*-pentanoylmannosamine (ManNPent) and *N*-hexanoylmannosamine (ManNHex) and we treated MCF7 breast cancer cells with those 5-acyl modified mannosamines. This is the strategy we refer to as metabolic glycoengineering (Figure 1). We found that applications of all non-natural sialic acid precursors led to decreased expression of Neu5Ac, reduced expression of polysialic acids (polySia) and in agreement with this finding, we observed reduced adhesion and migration. In addition, we examined the cell surface expression of the neural cell adhesion molecule (NCAM), and identified altered NCAM expression, which is accompanied by differential extracellular signal-regulated kinase (ERK) phosphorylation status.

## 2. Results and Discussion 

### 2.1. Cell Viability 

**Figure 2:**
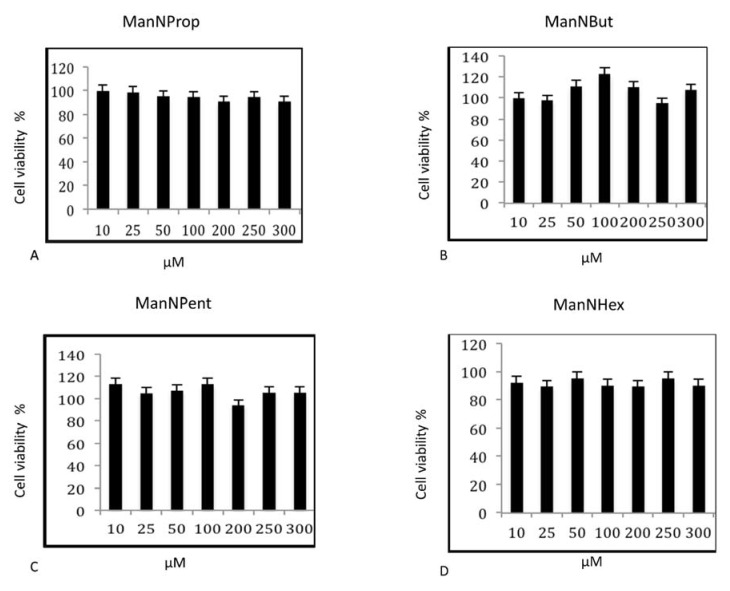
Cell Viability Assay. MCF-7 cells were cultured in the presence of various concentrations of non-natural Sia precursors for 48 h and assayed for cell viability by MTT assay. (**A**–**D**) represents the cell viability assay of MCF-7 cells treated with peracetylated mannosamine precursors. The bars represent mean values including standard deviations of four independent experiments.

In the first series of experiments, we tested the influence of non-natural Sia precursors on cellular toxicity. We incubated MCF7 cells with various concentrations of non-natural Sia precursors for 48 h and analyzed the cell viability by MTT (3-(4,5-dimethylthiazol-2-yl)-2,5-diphenyltetrazolium bromide), a tetrazole assay (Figure 2). There was no significant cell death observed after any precursor treatment of up to 300 µM. We therefore decided to use concentrations of 300 µM Sia precursors for all further experiments.

### 2.2. Analysis of Sia

**Figure 3:**
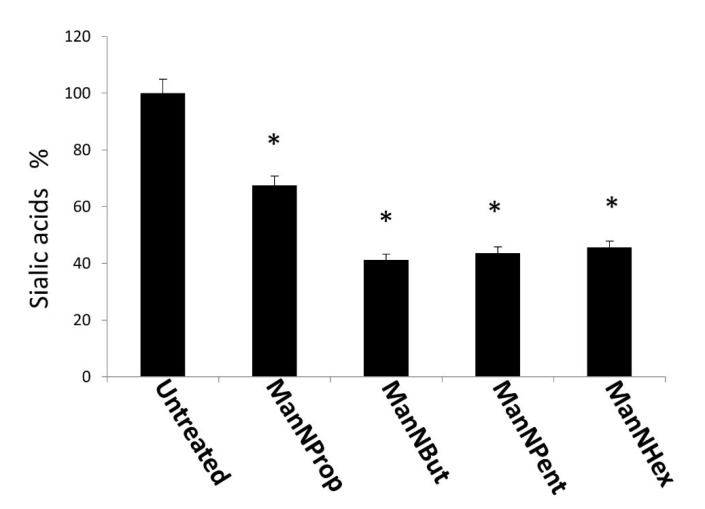
High performance liquid chromatography (HPLC) analysis of Sia. MCF-7 cells were cultured in the presence of 300 µM non-natural Sia precursors for 48 h and analyzed by HPLC. Sia (Neu5Ac) was quantified and expressed in percentage. The bars represent mean values including standard deviations of 4 independent experiments (* *p* < 0.001).

In a next experiment, we quantified and compared the expression of Sia in MCF-7 cells after culturing cells in the presence of 300 µM non-natural Sia precursors for 48 h. The relative percent of natural Sia was determined by high performance liquid chromatography (HPLC) after DMB-labelling (1,2-diamino-4,5-methylenedioxybenzene). Samples were injected and eluted with an isocratic solvent as described in the methods section. The peak corresponding to the retention time of the Neu5Ac standard was used for the calculation. *N*-propanoylmannosamine (ManNProp) treatment reduced the expression of natural Neu5Ac by up to 34% when compared with the untreated controls (Figure 3). ManNBut-treated cells displayed a 59% reduction in Neu5Ac (Figure 3, ManNBut). Moreover, when cells were engineered with ManNPent or ManNHex, Neu5Ac expression was reduced by up to 56% and 54%, respectively (Figure 3, ManNPent and ManNHex). In our previous work, we found a correlation between the length of the side chain at the 5-acyl position and sialylation or polysialylation [20]. Similarly in this study, we observed a gradual decrease of total Sia from ManNProp- to ManNHex-treatment. The acceptance of the non-natural Sia precursor as a substrate for Sia biosynthesis needs the activity of the key enzyme of the Sia biosynthesis—the GNE. Interestingly, GNE kinase tolerates unnatural mannosamines with a prolonged side chain up to ManNPent, but we observed a reduced natural Neu5Ac even when using ManNHex [21]. The incorporation of non-natural Sia in glycans of glycoproteins can also be regulated later in the pathway, during the CMP activation by the CMP-Sia synthase or on the level of the sialyltransferases. 

### 2.3. Lectin Analysis of GlcNAc

**Figure 4:**
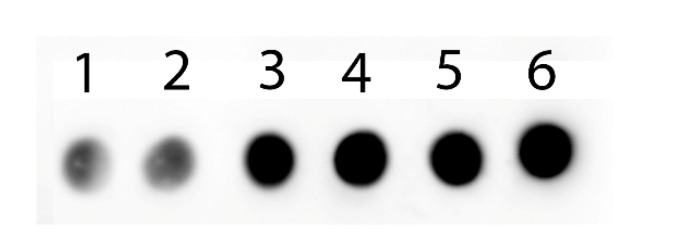
GlcNAc expression of MFC-7 cells in the presence of 300 µM non-natural Sia precursors. MFC-7 cells were supplemented with different modified sialic acid precursors and analyzed with succinylated wheat germ agglutinin (sWGA): 1-untreated, 2-ManNProp, 3-ManNBut, 4-ManNPent, 5-ManNHex.

Sialic acids mainly occupy the terminal position in glycans. A reduction in bound sialic acid in the presents of non-natural Sia precursors could lead to the exposure of underlying sugars like *N*-acetylglucosamine (GlcNAc). Therefore, we used succinylated wheat germ agglutinin (sWGA) to detect exposed GlcNAc in glycan structures. Terminal GlcNAc is increased in the presence of ManNBut, ManNPent and ManNHex, while no change was found in the presence of ManNProp when compared with the untreated cells (Figure 4). Interestingly, *N*-proponoyl mannosamine is better tolerated by the Sia biosynthetic pathway and produces *N*-proponoyl neuraminic acid which possibly masks the terminal GlcNAc, thereby possibly hindering sWGA binding to terminal GlcNAc. 

MCF-7 cells were cultured and treated with 300 μM non-natural Sia precursors for 48 h, and analyzed for the cells surface α 2,3 linked Sia using biotinylated Maackia amurensis lectin.

**Figure 5:**
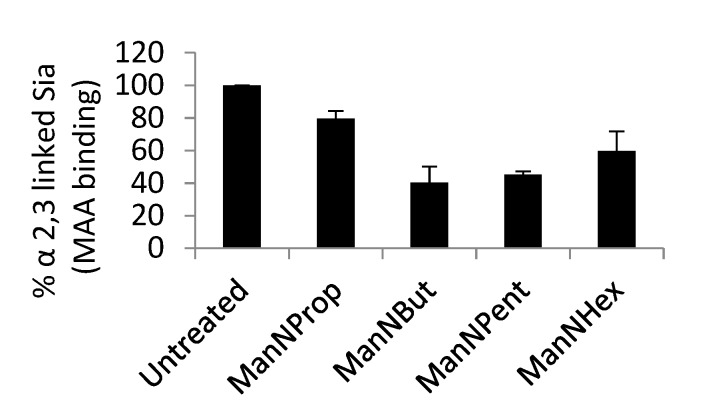
Flow cytometry analysis of cell surface α 2,3 linked Sia.

In order to examine the reduction in cell surface Sia, we used biotinylated MAA II lectin, which specifically binds to α 2,3 linked Sia in flow cytometry analysis. After Sia engineering with respective non-natural Sia precursors, the analysis of cell surface Sia demonstrates the reduction in α 2,3 Sia (Figure 5). We observed that ManNProp treatment reduces the overall cell surface α 2,3 Sia by up to 20%, whereas ManNBut treatment displays a dramatic 60% reduction in cell surface α 2,3 Sia. Interestingly, the ManNPent and ManNHex treatment reduces 55% and 40% of α 2,3 Sia.

### 2.4. PolySia and NCAM Analysis 

**Figure 6:**
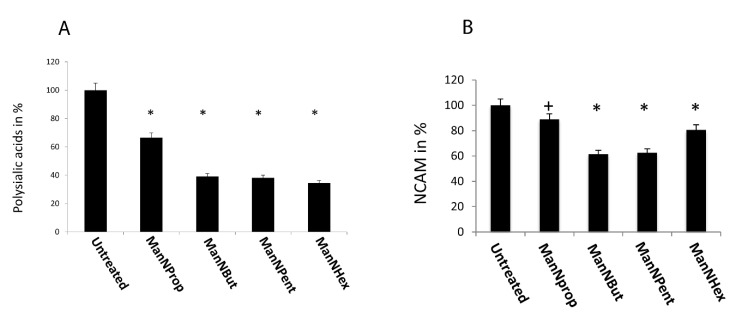
Cell surface expression of polySia and NCAM. MCF-7 cells were cultured in the presence of 300 µM of non-natural Sia precursors for 48 h and analyzed by flow cytometry using the 735 anti-polySia antibody. (**A**): PolySia expression in the absence of mannosamines was set to 100% and polySia expression in the presence of non-natural Sia precursors was expressed in percent of the control. The bars represent mean values including standard deviations of four independent experiments. (* *p* < 0.001). (**B**): NCAM expression in the absence of mannosamines was set to 100% and polySia expression in the presence of non-natural Sia precursors was expressed in percent of the control. The bars represent mean values including standard deviations of four independent experiments (+ *p* < 0.005, * *p* < 0.001).

As we have observed the changes in total Sia and α 2,3 linked Sia, we turned our attention towards polySia and its major carrier protein, NCAM. Cell surface polySia plays an important role in cell–cell or cell–matrix interaction and cell migration. Therefore, we analyzed the expression of polySia and NCAM by flow cytometry in MCF-7 cells. PolySia expression was reduced after glycoengineering with non-natural Sia precursor ManNProp by 33% (Figure 6A). Furthermore, glycoengineering with ManNBut resulted in a decrease of 60.8% in polySia compared to untreated cells, which indicates the significant inhibitory effect of ManNBut on polySia cell surface expression. Treatment of MCF-7 cells with ManNPent and ManNHex even led to a decrease in polySia by 62% and 65%, respectively. The underlying mechanism of this reduction might be explained by a reduction in affinity of the two polysialyltransferases ST8Sia2 and ST8Sia4 towards the non-natural Sia [22]. 

Since NCAM is the only carrier of polySia in MCF-7 cells, we also checked the expression of NCAM by flow cytometry after glycoengineering (Figure 6B). Culturing the cells in the presence of ManNProp for 48 h yielded a 11% reduction in cell surface expressed NCAM, while after ManNBut and ManNPent treatment, we measured a reduction of nearly 40% in NCAM expression, which could partially be responsible for the 60% reduction in polySia under the same conditions. However, engineering with ManNHex displayed only a 20% reduction in cell surface expressed NCAM. This reduction in NCAM expression after glycoengineering could be explained by the turnover as well as the half-life of NCAM, since these are dependent on polysialylation [23]. Furthermore, we recently observed reduced NCAM expression after treatment of cells with fluorescent CMP-Neu5Ac mimetics, which interfere with sialyltransferases and also reduce polysialylation of NCAM [24]. Thus, the reduction in cell surface expression of NCAM could be due to the reduced half-life of NCAM.

### 2.5. ERK Phosphorylation Status Analysis

**Figure 7:**
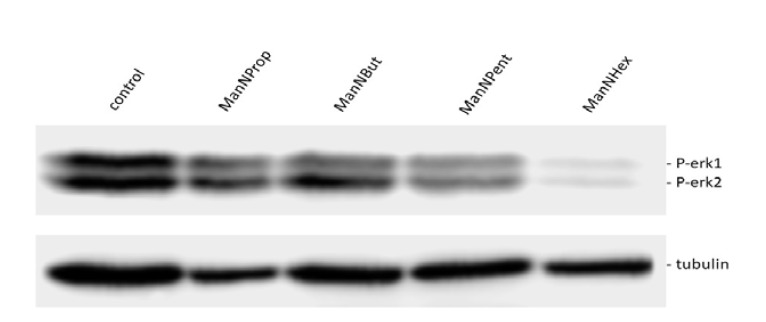
ERK phosphorylation. MCF7 cells were cultured for 48 h in the presence of 300 µM of mannosamine derivatives. Cell lysates were prepared and immunoblotted with corresponding phospho ERK (PERK1 and 2) antibody. The blot was stripped and reprobed for beta tubulin protein as a loading control.

The observation of the reduction in cell surface polysialylation and NCAM expression after glycoengineering prompted us to analyze the ERK phosphorylation (Figure 7), since ERK activation is involved in many cell adhesion molecule-dependent signal transduction pathways [25,26]. We observed a significant reduction in ERK1-phosphorylation after culturing MCF7-cells with ManNProp or ManNBut, but only a slight reduction in ERK2-phosphorylation. However, treatment with ManNPent and ManNHex drastically reduced phosphorylation of both ERK1 and ERK2. PolySia significantly interferes with NCAM-mediated interactions. We observed reduced sialic acid and cell surface polysialic acid, and this correlates with the reduction in ERK1 and ERK2 phosphorylation. The consequence of ERK phosphorylation depends on environmental cues and the crosstalk of other signaling pathway initiating cell differentiation, proliferation, migration or death based on the growth factors, cytokines or mitogens [27,28]. For example, treatment of neuroblastoma cells with endoN reduces polySia and significantly reduces ERK phosphorylation [29]. However, culturing PC12 cells in the presence of ManNProp activates ERK phosphorylation and promotes neurite outgrowth [30]. Thus, interfering with natural sialylation and the incorporation of non-natural Sia is a possibility to modulate cell signaling, and it may also regulate gene expression through various channels. Sia engineering reduces natural Sia during biosynthesis by (I) competing with the natural Sia precursor ManNAc as a substrate; (II) inhibiting the kinase activity of the GNE. The inhibition of natural Sia can also occur by steric hindrance due to the presence of non-natural sialic acid at the acceptor site. Altogether, this affects the biological and biochemical property of the associate proteins. Most of the cell adhesion and cell surface proteins are sialylated, and reduced natural Sia alters the protein localization as well as the protein–protein interaction, which subsequently affects the cell signaling. As a consequence of the altered cell signaling, the gene expression is also changed. This may lead to an overall effect on cell behavior and function.

### 2.6. Functional Assays

#### 2.6.1. Cell Adhesion 

**Figure 8:**
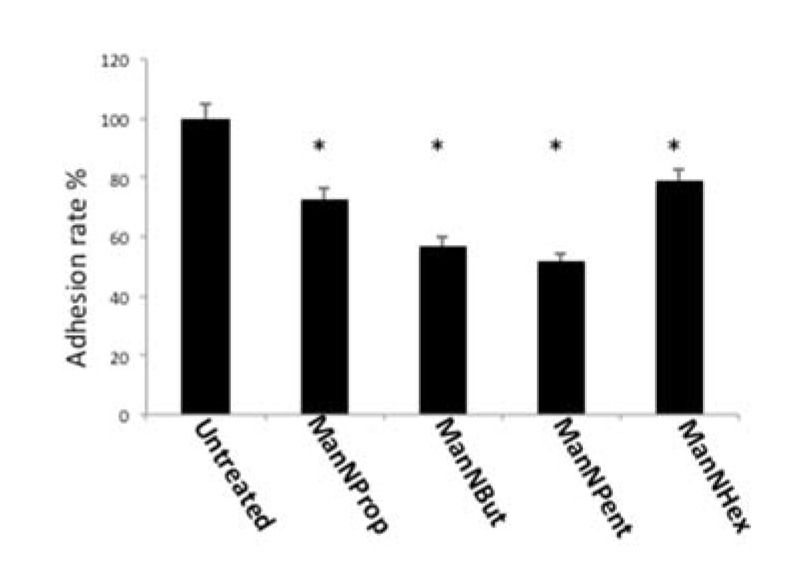
Cell adhesion. MCF-7 cells were cultured in the presence of 300 µM of non-natural Sia precursors for 48 h and cell adhesion was quantified using a real time cell analyzer. Cell adhesion in the absence of mannosamines was set to 100% and adhesion in the presence of non-natural Sia precursors was expressed in percent of this control. The bars represent mean values including standard deviations of four independent experiments. (* *p* < 0.001).

Sia is known for its antiadhesive properties due to its negative charge and thereby interferes with protein–protein interactions, as shown by various studies [31,32]. We therefore quantified cell adhesion to laminin of MCF-7 cells, which were cultured in the absence or presence of non-natural Sia precursors, and we found a significantly reduced cell adhesion (Figure 8). Treatment with ManNProp resulted in a 27% reduced adhesion; however, ManNBut or ManNPent, had a much stronger potency to interfere with cell adhesion (43% and 48%, respectively). Incubation with ManNHex decreased cell adhesion only by 22%. 

The ability of cancer cells to modulate adhesion is the prerequisite to establish cancer cell population at their metastatic site. Although Sia has been considered to be an antiadhesive molecule, it also acts as a ligand for selectins at the metastatic site for primary attachment. Glycoengineering with non-natural Sia precursors reduces the natural Sia and reduces cell adhesion to the extracellular matrix protein laminin in this context. Since we observed a significant reduction in natural total Sia and polySia after glycoengineering with non-natural Sia precursors, the incorporation of non-natural Sia could be responsible for the reduced cell adhesion to laminin. The incorporation of non-natural Sia after glycoengineering has been demonstrated in a variety of studies [17]. In addition, previous studies suggest that non-natural polySia could be a possible explanation for reduced binding and reduced neurite outgrowth of Hela cells, ectopically expressed with NCAM and polysialytransferases [33]. Recent investigations on inhibiting Sia expression using fluorinated CMP Sia-mimetic have shown a dramatic reduction in the cell surface sialylation and reduction in cell adhesion to collagen I in two different cancer cell lines [34]. This could suggest that primary tumor cells and metastatic cancer cells not only vary in the Sia expression, but also could differ in Sia linkages to provide ligands for the metastatic niche. 

#### 2.6.2. Cell Migration 

**Figure 9:**
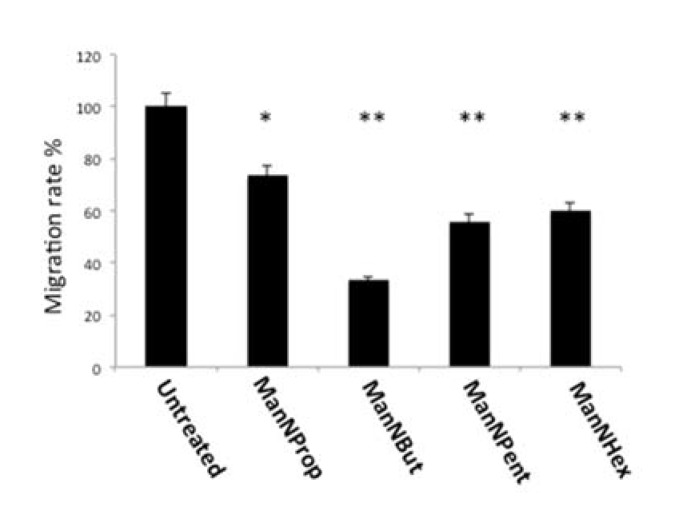
Cell migration. MCF-7 cells were cultured in the presence of 300 µM of non-natural Sia precursors for 48 h and cell migration was quantified using a real time cell analyzer. Cell migration in the absence of mannosamines was set to 100% and migration in the presence of non-natural Sia precursors was expressed in percent of this control. The bars represent mean values including standard deviations of four independent experiments (* *p* < 0.005, ** *p* < 0.001).

Sia and polySia are known to participate in the migration of metastatic cancer cells [35,36]. In general, increased expression of polySia leads to increased migration. We therefore performed migration assays using MCF-7 cells treated with non-natural Sia precursors. In general, migration of glycoengineered cells was reduced in comparison with untreated controls (Figure 9). When comparing the individual precursors, ManNProp treatment led to a reduction in migration by 27%, whereas ManNBut displayed a reduction in migration of up to 67%. ManNPent and ManNHex reduced migration by 44% and 40%, respectively. Migration properties of the cancer cell have been at the forefront in regard to the examination of metastasis. Epithelial to mesenchymal transition (EMT) is the classical mechanism during cancer progression. During the initial phase of EMT, several epithelial cell surface glycoproteins like MUC1 and E-cadherin are down regulated, but gain N-cadherin and integrin after being transitioned to the mesenchymal cell type [37]. All cell surface glycoproteins of both the epithelial and mesenchymal types were sialylated. Since glycoengineering using non-natural Sia precursors reduces the natural Sia content significantly, this impacts the biological properties of the Sia-modified glycoproteins. As migration per se involves both extra- and intra-cellular events, reduced sialylation could primarily interfere in the biological functions that are critically involved in mediating migration. Integrins are associated with migration and invasion; studies have shown that alpha 2,6 linked Sia on integrins alters its confirmation, and as a result, it enhances the binding to the collagen 1 [35]. Furthermore, it enhances the binding of talin to the cytoplasmic part of the integrin and thereby promotes migration [36]. Another study demonstrates that the sialyltransferase ST6Gal1 increases alpha 2,6 linked Sia on integrin and elevates the migration property via activating the focal adhesion kinase (FAK) pathway [35]. Taken together, we present novel data that clearly demonstrate the role of Sia during cancer progression and also offer a possibility to interfere in this process by water-soluble small Sia precursors. 

## 3. Materials and Methods 

### 3.1. Chemicals and Reagents

The D-mannosamine hydrochloride, the anhydride form of *N*-propanoyl, *N*-butanoyl, *N*-pentanoyl, *N*-hexanoyl, sodium methoxide, pyridine, acetic anhydride, TLC plates and the respective chromatographic solvents such as chloroform and methanol were purchased from Sigma Aldrich, synthesis and analytical grade quality (Hamburg, Germany). MCF-7 cells purchased from DSMZ (Braunschweig, Germany). Cell culture medium, Dulbecco’s modified Eagle medium, serum, penicillin, and streptomycin were purchased from Gibco (Darmstadt, Germany). Laminin was purchased from Sigma Aldrich (Hamburg, Germany) and E and CIM (cell invasion/migration) plates were purchased from OLS (Bremen, Germany). Protease and phosphates inhibitor were purchased from Sigma Aldrich (Hamburg, Germany). 735 anti-polySia antibody was kindly gifted by Prof. Dr. Rita-Gerady-Schahn (Medizinische Hochschule Hannover, Germany). 123C3 anti-NCAM and pERK 1 and 2 were purchased from Cell Signaling Technology Europe B.V, (Frankfurt, Germany) and beta tubulin antimouse antibody was purchased from Thermofisher (Darmstadt, Germany). Biotinylated Maackia amurensis lectin II, and biotinylated wheat germ agglutinin were obtained from vector labs (Curlingame, CA, USA).

### 3.2. Synthesis of Non-Natural N-acylmannosamines

Mannosamine derivatives (*N*-propanoyl, *N*-butanoyl, *N*-pentanoyl, *N*-hexanoyl) were prepared in our laboratory and peracetylated. Briefly, 10 mM of mannosamine hydrochloride was dissolved in 30 mL of methanol and kept in ice, followed by the addition of 12 mM of respective carbonic acid anhydride. This was then stirred for 2 h in ice and the reaction was dried in a vacuum evaporator and checked by TLC (thin layer chromatography) analysis. The product was then purified by silica gel 60 column chromatography using acetic acid ethylester/methanol/water at 5:2:1 to 10:2.1 based on the polarity of the anhydride [18,38]. The purity of the synthesized product was controlled by mass spectrometry analysis. In order to increase the cellular uptake of mannosamine derivatives, the *N*-acyl-d-mannosamine derivatives were peracetylated by the addition of the required amount of 100 µL acetic anhydride to 300 µL of pyridine medium and incubated overnight at room temperature. TLC analysis was then carried out, followed by the medium being purified by silica gel column chromatography [19].

### 3.3. Cell Viability Assay by 3-(4,5-Dimethylthiazol-2-yl)-2,5 diphenyl tetrazolium bromide (MTT)

Cells (10,000/well) were seeded in 96 well plates and treated with various concentrations (0–300 µM) of ManNProp, ManNBut, ManNPent, and ManNHex for 48 h. Cultured cells were replaced with fresh medium every 24 h. After 48 h, media were aspirated and replaced with 200 µL of fresh medium, to which 20 µL of 5 mg/mL of MTT reagent was added and incubated at 37 °C for 4 h. Then media were aspirated and 150 µL of DMSO was added to each well. The plates were kept in a plate shaker for 20 min for solubilization of the formazan crystals, and this was followed by the measuring of the absorbance at 560 nm in the ELISA (enzyme-linked immunosorbent assay) plate reader (Thermo Scientific Multiskan EX Langenselbold, Germany).

### 3.4. Cell Culture and Glycoengineering of MCF-7 Cells

MCF7 cells were cultured in DMEM (Dulbecco’s modified Eagle’s medium) containing 10% FCS (fetal calf serum) with L-glutamine, penicillin and streptomycin. Cultured cells were glycoengineered by treatment with 300 µM peracetylated non-natural Sia precursors (ManNProp, ManNBut, ManNPent and ManNHex) for 48 h. Cells were washed with PBS (phosphate-buffered saline) and dissociated with trypsin EDTA (ethylenediaminetetraacetic acid) followed by neutralization with DMEM medium containing 10% FCS. Cells were further washed with PBS and pelleted by centrifugation at 0.1× *g* for 5 min for further analysis. 

### 3.5. HPLC Analysis of Sia

MCF-7 cells were glycoengineered for 48 h, and cells were dissociated and pelleted by centrifugation. Cell pellets were homogenized and followed by mild acid hydrolysis of Sia with an equal volume of 4 M propionic acid at 80 °C for 4 h. After hydrolysis, the total lysate was cooled in ice for 10 min, followed by centrifugation at 14,200× *g* for 20 min at 4 °C. The supernatant was subjected to centrifugal ultrafiltration at 13,000× *g* for 20 min at 4 °C. The flow through was carefully collected, freeze-dried and lyophilized overnight. The samples were dissolved in 100 µL of water to which equal volume of DMB reagent was added and incubated at 50 °C for 2.5 h. Samples were cooled briefly in ice followed by short centrifugation at 10000× *g* for 1 min. 20 µL of DMB labeled samples were injected into the HPLC column and eluted the sialic acids using isocratic solvent, acetonitrile/methanol/water at 8/6/86 ratios at a flow rate of 0.6 mL/min. Neu5Ac standard labeled with DMB was used as a reference.

### 3.6. Lectin Staining

For dot blot analysis, lysates from untreated, ManNProp-, ManNBut-, ManNPent- and ManNHex-treated cells were processed with a 5x protein sample buffer. A total of 5 μg of protein (5 μL) was added to the nitrocellulose membrane and allowed to air-dry. Afterwards, the nitrocellulose strips were blocked with 3% BSA (bovine serum albumin) in TBS (tris-buffered saline) for 1 h at room temperature. In addition, the membranes were incubated with Tris buffer saline contains tween 20 (TBST) containing 1:1000 biotinylated, succinylated wheat germ agglutinin lectin for 3 h at room temperature. This was followed by three successive washes with TBST for 10 min each. To this, membrane HRP(horseradish peroxidase)-conjugated streptavidin at 1:20,000 in TBST was added and incubated for 1 h. Finally, these membranes were again washed with TBST 3 times. This was followed by the development of the lectin blot by a chemiluminescence substrate reagent. 

For flow cytometry analysis, cells were cultured in the standard 6 well plates and treated with non-natural sialic acid precursors for 48 h. Further cells were washed twice with ice cold PBS followed by dissociation of the cells with trypsin/EDTA. Again, cells were washed once with 500 μL FACS (fluorescence-activated cell sorting) buffer (PBS containing 2% FCS) and centrifuged. The cell pellet was resuspended in 100 μL of FACS buffer containing biotin-conjugated Maackia amurensis lectin II (B-1265-1,1:100 dilution) for 30 min in ice under dark conditions. After this, cells were further washed twice with FACS buffer, followed by the cell pellet being suspended in 100 μL fresh FACS buffer containing streptavidin, DyLight 488 (SA-5488-1,1:100 dilution) and incubated for 20 min in ice under dark conditions. Once again, the cells were washed twice with FACS buffer and further resuspended in fresh FACS buffer. The measurements were made using a BD caliber flow cytometer (BD biosciences, Heidelberg, Germany) according to the manufacturer’s instructions.

### 3.7. PolySia and NCAM Analysis by FACS 

Cells were seeded in the 6 well plates and engineered with Sia precursors for two days. Cells were washed with ice-cold PBS and disassociated with trypsin/EDTA. Disassociated cells were washed twice with PBS and incubated with a 1:100 dilution of anti-polySia antibody in 100 µL of RPMI-1640 (Roswell Park Memorial Institute) with 5% FCS for 60 min. For the NCAM analysis, cells were incubated with anti-NCAM antibody at 1:100 dilution in PBS containing 2% FCS. Subsequently, cells were washed twice with 0.5 mL of ice-cold PBS, followed by the addition of antimouse secondary antibody (Dianova GmbH, Hamburg, Germany) conjugated with fluorophore Dylight 488 (1:100). Cells were then incubated on ice for 45 min and washed twice with 0.5 mL of ice-cold PBS. Following this, 600 µL of fresh ice-cold PBS was added to the pelleted cells. Measurements were made using a BD caliber flow cytometer (BD biosciences, Heidelberg, Germany) according to the manufacturer’s instructions. 

### 3.8. Immunoblotting 

Cells were seeded in the 6 well plates and cultured for 48 h with the respective mannosamine derivatives. Fresh medium containing mannosamine derivatives was replaced every 24 h. After 48 h, cells were washed with PBS, dissociated with trypsin EDTA and neutralized with 5% serum-containing medium. Cells were washed once again with PBS and pelleted by centrifugation. Cell pellets were lysed and solubilized with a RIPA (radioimmunoprecipitation assay) buffer containing protease and phosphatase inhibitors. A total of 50 micrograms of protein was loaded on the 10% SDS PAGE gels and separated at 80 V for 3 h. Proteins were transferred onto nitrocellulose membranes and blocked with TBS containing 5% BSA for 1 h at room temperature (RT). Blots were incubated with anti-pERK1 antibody at 1:1000 dilution or anti-beta-tubulin antibody at 1:5000 dilution overnight at 4 °C. Blots were washed 3 times with TBS-Tween (TBST) and incubated with HRP-conjugated antimouse secondary antibody in TBST containing 3% milk for 1 h at RT. Again, blots were washed a further 3 times with TBST for 10 min each. pERK 1 or 2 bands were developed using a chemiluminescence reagent and detected by Chemidoc XRS system (Bio-Rad Laboratories GmbH, München, Germany).

### 3.9. Adhesion Assay

E-plates were coated with laminin protein at 20 µg/mL concentration and incubated for 60 min at 37 °C. An appropriate number of glycoengineered cells was added to the E-plate and they were allowed to settle. Afterwards. the E-plate was kept in the station and started to measure the adhesion by monitoring the impedance every 5 min for 4 h.

### 3.10. Migration Assay

Cells were glycoengineered with sialic acid precursors for 48 h, washed twice with PBS and dislodged with a PBS/EDTA buffer, and washed once with serum-free medium. Cells (50,000/well) were added to the upper chamber of the 16-well CIM plate (OLS xCELLigence, Bremen, Germany). The lower chamber had been previously filled with 160 µL of complete medium. After the cells were settled in the upper plate (kept in the cell culture hood for 30 min), the CIM plate was placed in the station (RTCA, OLS xCELLigence, Bremen, Germany). Migration of the cells was monitored by measuring the impedance every 15 min for 24 h.

## 4. Conclusions

Glycoengineering MCF7 cells with non-natural Sia precursors reduced the total and polySia significantly. Due to the reduction in Sia, the sialic-acid-engineered cells display exposed terminal GlcNAc as well as reduced adhesion and migration. Among the non-natural Sia precursor treatment, ManNBut and ManNPent interfere significantly with biological properties of the engineered cells. We also observed reduced cell surface NCAM, which could potentially be involved in the interference of biological properties. Furthermore, we observed that engineering with non-natural Sia precursors reduces ERK phosphorylation. Thus, it is confirmed that modulation of natural sialic acids by this approach induces complex cellular changes, which involves reduced cell surface NCAM possibly as a result of reduced NCAM half-life or altered localization due to reduced polySia, which needs further investigation. Furthermore, changes in ERK phosphorylation associated with reduced adhesion and migration suggests that Sia is involved in this process via modulating protein property, that is, altering cell signaling which leads to changes in the biological behavior of the cell. Thus, our study provides a strong basis for the further investigation in the perspective of sialic acid engineering as a biological toolbox for investigating the role of Sia in breast cancer progression and metastasis.
